# Miscellaneous Intelligence, &c. &c.

**Published:** 1824-03-01

**Authors:** 


### XII.
### INTELLIGENCE, &c.

M. Geoffroy St. Hilaire in Paris, and M. Jean Mueller of Leipzick, have announced Memoirs, yet in embryo, on the *Respiration of the Fœtus in Utero*. These discoveries will puzzle our writers on infanticide!

A case of imperforate anus and urethra is now to be seen in the person of a mendicant near the village of Nancy, in Lorraine. The patient vomits his excrements and urine. The details will be published in the *Archives Generales.*

In the space of five years, (from the 1st January, 1817, till the end of December, 1821) 1730 cases of suicide have occurred in Paris. Of these, 1124 were males, and 606 females—862 were married, and 868 unmarried. The causes were as follows:—

| | |
|---|---|
| 1. Love | 121 |
| 2. Diseases, disgust of life, alienation, domestic quarrels | 628 |
| 3. Gaming, lotteries, debauches, &c. | 228 |
| 4. Indigence, and deranged affairs | 342 |
| 5. Fear of punishment and blame | 58 |
| 6. Motives undiscovered | 353 |
| Total | 1730 |

*Archives Generales, November*, 1823.

### DOSES OF MEDICINE.

Much misunderstanding of the word "dose" has prevailed in this country respecting the continental practice. *Here* it signifies *what* is taken of a *medicine* at one time—on the *continent*, it means what is taken of a medicine in 24 hours. The large doses of medicine exhibited by our Italian brethren are thus, in part at least, accounted for. But it must be remembered, that the Italian physicians exhibit large doses of medicine (as antimony for instance) in *acute* diseases, as a substitute for venesection. It is certain that, in such circumstances, medicines have not one tenth the effect which they have in health, or where free depletion has preceded their exhibition.

### OPERATIVE CHEMISTRY.

In the next number of this journal, (being the first of a new series) we shall dedicate a portion of our extra-limites department to a paper by Mr. Battley on opium. In the succeeding numbers, a series of papers on Practical Subjects of Pharmaceutical Chemistry, are expected from the same pen.

*Literary Notice.* Dr. Forbes of Chichester, has in the press a work on the Diagnosis of Thoracic Diseases, which will comprise, 1. A Condensed Translation of Avenbrugger's Treatise *on Percussion of the Chest*, with Corvisart's Commentaries; 2. A Translation of the Diagnostic Part of Laennec's Treatise *on Mediate Auscultation;* and 3. A Series of Original Cases (with dissections) illustrative of the Utility of Percussion and the Stethoscope in the Diagnosis of Diseases of the Chest.

*Ligature of the Arteria Innominata.* It has surprised us a little to see all our cotemporaries employed in detailing Dr. Valentine Mott's Operation for Aneurism of the Arteria Innominata, or Subclavian, which, occupying four pages, was published in this Journal between four and five years ago, viz. in our number for July 1819, p. 147, *et seq.* It has still more surprised us that it has escaped our cotemporaries, that it was very far from satisfactorily proved that there was any aneurism in the case. A furious paper war was carried on in America some years ago respecting this case, rendering it still more remarkable that it should now be brought up as new.

### MEDICO-CHIRURGICAL REVIEW.

The editor respectfully informs various private friends, public societies, and editors of Journals, that his avocations entirely prevent him any longer forwarding to them the numbers of this Journal. The various foreign and domestic Journals are ordered by him to be purchased for this Review ; and those who think a reciprocal return desirable will follow his example.

---

### Mr. COLE'S TRUSS.

I hereby certify, that I have worn the trusses of Mr. Cole's invention for sixteen months past, and have found them more easy and more efficacious than any others which I had worn for fourteen years previously.

*London, Sept.* 1823. CARYER VICKERY, Surgeon, R.N.

---

London to Wit.—Robert Hubbard of the Parish of St. Sepulchre, London, maketh oath and saith, that he hath laboured under a very severe rupture upwards of thirty years, which has increased to an enormous size, and disabled him for many years past from following his avocation as a smith. This deponent likewise worked a journeyman for several eminent truss makers in London, but he never saw a truss in his life which was calculated to keep up his rupture, (in its present state) until Mr. Cole, of London Bridge, applied one of his invention, whereby he the said deponent hath been rendered perfectly secure and free from pain during eighteen months past, and that he is thereby enabled to work at his business, or walk four miles in one hour with ease. ROBERT HUBBARD.

20th *August*, 1323. Sworn at the Mansion House, London, before me W. HEYGATE, Mayor.

---

I hereby certify, that I have suffered twice the dangers of strangulated hernia, which had almost proved fatal. I placed myself under the care of an eminent surgeon in this neighbourhood, from whom I learned that Mr. Cole had given proofs of extraordinary talent in his profession ; a truss was immediately procured which effected a pressure of at least sixteen pounds upon the hernia, which was not removed from the part for fourteen days and nights. He has since applied a bandage, which I have worn for a much longer period, without removing it. It is with great satisfaction that I can inform those who are similarly circumstanced, that I have never suffered a shadow of pain since I have been under his care.

*Dover Castle, Inn,* JOHN MARTIN.
*21st November*, 1822. Licenced Victualler, Deptford.

---

### GAZETTING.

In consequence of the death of Dr. J. Johnson of the Albany, a pretty general idea prevailed, and even yet prevails, among his friends in the country, that the Editor of this Journal has gone to the shades. As an episode to this delusion, it was last month *gazetted* by the "Southcoate sage," that Dr. Johnson *had gone* to Paris for *surgical assistance*, and was therefore only the *nominal* editor of this Review ! Now this journey to Paris for surgical assistance was, in reality, a tour of about 2500 miles through France, Switzerland, Germany, and Belgium, in the months of August, September, and October last—during which tour Dr. Johnson never took a dose of physick, and never enjoyed robuster health or higher spirits in the whole course of his life.

It has been frequently asked, how it happened that Dr. Johnson was honoured with such an unusual proportion of vituperation in the "GAZETTE OF HEALTH"—or more properly speaking, the GAZETTE OF FILTH, conducted by the renowned Æsculapius of the Egyptian Hall—Richard Reece, M.D. (which signifies, Dealer in Medicine Chests) of the University of London ; Physician-Accoucheur to Her late Holiness, Johanna Southcoate, &c. ? There are reasons for all things—and for *this* there happens be no less than *ninety* very weighty ones. About ten or eleven years ago, the Piccadilly M.D. or drug-dealer, conceived the benevolent project of extending the benefits of his *disinterested* advice to our Indian possessions ; (instead of confining them to the Medical Hall and Bolton Row) and with that view, published, by the aid of a *Gazetteer*, or rather GARRETTEER, a work entitled—"The Medical Guide for Tropical Climates," in which, with laudable zeal, were well puffed off the Medicine Chests, Rhatany Root, "*Preventive Soap*," Buchu Leaves, &c. &c. to be found in genuine perfection *only* at the Medical Hall in Piccadilly. Who would have thought that there was any harm in this?—especially when the work was dedicated, "BY PERMISSION," to Dr. William Dick, Principal Physician to the East India Company—and not only *by permission* for the dedication, but as was pointedly averred, with the entire *approbation of the work itself*, by that distinguished physician? In the face of all this, a review appeared in one of the Medical Journals soon afterwards, portraying so felicitously the matchless absurdity, quackery, and ignorance of the publication, that it fell still-born from the press amidst a burst of ridicule. This was the least of the bad consequences attending this wicked critique. Dr. William Dick, the patron and approver of the work, immediately came forward, and affirmed that, not only did he never lend his name to such a wretched production, but instead of *approving* it, *he never saw or heard of it*, till the just critique of it appeared in the Journal—thus affixing an indelible stain on the moral character of an author who could deliberately publish a falsehood in the title page, and another falsehood in the preface of his book —and both for the purpose of puffing off his merchandize!!*

Our readers may form some idea of the paroxysm of rage produced by this public detection of unparalleled effrontery aud falsehood. There was no resource but a prosecution, *by indictment*, of the editors and publishers of the critique. A bill was accordingly filed, and great preparations were made; but, lo! when the day of trial came, the heart of the "Southcoate Sage" failed, and, rather than let the transaction come into court, he pocketed the loss of his character for veracity—the edition of his Tropical Guide—and *ninety guineas* law expences!—A pill so bitter to swallow and hard to digest, has, ever since that period, occasioned a monthly effusion of gall against Dr. Johnson, the supposed writer of the critique. Considering the nature and extent of the offence, Dr. J. does not wonder at, nor complain of, the continued and unextinguished ire of the "Southcoate Sage." He has inflicted on that worthy personage a wound, the smarting of which can never cease on this side of the grave—and consequently his share of vituperation must, in common justice, be greater than that of all others put together. His friends have now a key to the GAZETTE OF FILTH—and can explain to "the clergy and heads of families" in the country, the secret springs which actuate that *disinterested* publication. We are thoroughly convinced that, in every instance where its reptile editor has squirted his filth on the members of the profession, from the College of Physicians down to the Sage's own fraternity of Quacks, the same base and dastardly personal motives were at the bottom of the transaction, could they be brought to light.† In our next number, we promise our readers a hearty laugh at the portraiture (drawn faithfully from the writings and actions of the "Southcoate Sage") which this disgrace to the very dregs of medical society presents of absurdity, impudence, baseness, and mendacity.

---

DR. DICK'S LETTER.

"* Gentlemen.---You have severely censured me for approving of a book entitled "The Medical Guide for Tropical Climates," by Richard Reece, M.D. and I must acknowledge, that you could not condemn me too much, had there been any truth in the charge. But I most solemnly declare, thst I did not see a single line in the said book *before or after* its publication; nor did I know that it existed, till I read your just criticisms on it. *New Med. Journal*, 1814. WILLIAM DICK."

† To his utter disgrace, *one physician* in this metropolis, whose birth, parentage, and academical education, *ought* to have taught him to respect himself as well as his profession, has had the meanness, and the wickedness to write two or three *anonymous* letters in the infamous publication abovementioned; and that for the sole purpose of traducing the character of some of his brethren, whose talents, integrity, and success were greater than his own!---He is now meeting *his* deserts---sinking fast into ruin and contempt.

